# 

**Published:** 1886-04

**Authors:** 


PAGE

ORIGINAL COMMUNICATIONS.—Rabies in the Dog in its Ralation to Rabies in Man. With Especial Reference to its Prevention, and to Pasteur's System of Preventive Inoculation. Frank S. Billings, D.V.S. .... 161
- Pasteur and his Work .... 190
- Osteo-Porosis. H. F. James, V.S .... 193
- The Differential Patho-Anatomical Diagnosis in Glanders. Prof. Schutz .... 196
- Venereal Disease in the Lower Animals. Prof. Thomas Walley .... 213
- Typhoid Fever in Animals. J. Bland Sutton, M.D .... 218

EDITORIAL DEPARTMENT .... 224
CASE DEPARTMENT .... 233
PROCEEDINGS VETERINARY MEDICAL SOCIETIES .... 235
PROGRESS VETERINARY SCIENCE .... 236
OBITUARY .... 237
REVIEWS .... 238

